# Soil-Transmitted Helminths in Tropical Australia and Asia

**DOI:** 10.3390/tropicalmed2040056

**Published:** 2017-10-23

**Authors:** Catherine A. Gordon, Johanna Kurscheid, Malcolm K. Jones, Darren J. Gray, Donald P. McManus

**Affiliations:** 1 QIMR Berghofer Medical Research Institute, Molecular Parasitology Laboratory, Queensland 4006, Australia; Don.McManus@qimrberghofer.edu.au; 2 Australian National University, Department of Global Health, Research School of Population Health, Australian Capital Territory 2601, Australia; Johanna.Kurscheid@anu.edu.au (J.K.); darren.gray@anu.edu.au (D.J.G.); 3 School of Veterinary Science, University of Queensland, Brisbane, QLD 4067, Australia; m.jones@uq.edu.au

**Keywords:** soil-transmitted helminths, *Trichuris trichiura*, *Ascaris lumbricoides*, hookworm, *Ancylostoma ceylanicum*, *Strongyloides stercoralis*, South East Asia, Australia

## Abstract

Soil-transmitted helminths (STH) infect 2 billion people worldwide including significant numbers in South-East Asia (SEA). In Australia, STH are of less concern; however, indigenous communities are endemic for STH, including *Strongyloides stercoralis*, as well as for serious clinical infections due to other helminths such as *Toxocara* spp. The zoonotic hookworm *Ancylostoma ceylanicum* is also present in Australia and SEA, and may contribute to human infections particularly among pet owners. High human immigration rates to Australia from SEA, which is highly endemic for STH *Strongyloides* and *Toxocara*, has resulted in a high prevalence of these helminthic infections in immigrant communities, particularly since such individuals are not screened for worm infections upon entry. In this review, we consider the current state of STH infections in Australia and SEA.

## 1. Introduction

Soil-transmitted helminths (STH) are estimated to infect 2 billion people worldwide. Many of these infections occur in South-East Asia (SEA) [1,2]. Species included in the term STH are the human hookworm species *Ancylostoma duodenale* and *Necator americanus*, the human roundworm *Ascaris lumbricoides*, and the human whipworm *Trichuris trichiura* [3]. Hookworm and *Trichuris* have zoonotic counterparts (*A. caninum, A. ceylanicum, T. suis,* and *T. vulpis*) [4,5,6,7,8,9,10,11,12,13,14]. *A. lumbricoides* itself is a zoonosis as the previously-identified pig roundworm, *A. suum*, has been found through molecular characterisation to be nearly identical to *A. lumbricoides,* and instead represents a haplotype of *A. lumbricoides* [6,15]. *Toxocara canis* and *Strongyloides stercoralis* are additional important nematode species of dogs that can also infect humans and are included in this review. *Strongyloides* is estimated to infect 30–100 million people [16,17], while the seroprevalence (2%–5% in urban areas, 14.2%–37% in rural areas) of *Toxocara* in developed countries indicates that the number of people at risk of infection may be in the millions [18]. While *Toxocara* and *Strongyloides* have low prevalences in Australia, our closest neighbours in SEA, as well as many countries from which refugees and immigrants originate, are highly endemic for these parasites. These helminths are also considered neglected tropical diseases (NTD) due to their occurrence in low socio-economic regions and have thus not received as much attention as other diseases occurring in developed countries. Primarily, these helminthic infections are endemic in tropical and subtropical areas due to the requirements for warm moist soil for egg or larval development.

Another potential reason for their status as NTDs is the chronic rather than acute nature of the infections they cause. Symptoms are similar between the causative species and are generally non-specific; namely nausea and/or vomiting, diarrhoea, abdominal pain, and fever. In adults the impact can be seen as lower ability to work. As such, the impact of these parasites should be examined by considering the disability adjusted life years (DALYs) to measure disease burden. According to the WHO definition, one DALY is one year of life quality lost compared to a healthy individual, and the sum of these DALYs across a population is a measurement of the gap between current health status and an ideal health situation [19]. 

As of 2010 there were an estimated 438.9 million people infected globally with hookworm, 819.0 million with *A. lumbricoides,* and 464.4 million with *T. trichiura* [20]. It was calculated that STH contributed to 4.98 million years lived with disability (YLDs), with 65% attributed to hookworm, 22% to *A. lumbricoides,* and 13% to *T. trichiura* [20]. The DALYs for intestinal helminths (including only *A. lumbricoides, T. trichiura,* and hookworm) have been reduced from 170 per 100,000 (94–290) in 1990 to 75 per 100,000 (43–128) in 2010 and 69.4 per 100,000 (43.3–106.4) in 2013 [21,22]. DALYs are not considered a good measurement of the burden of disease for *S. stercoralis* since the majority of infections cause limited clinical symptoms; the most common complaint and symptom is stomachache. Poor diagnostics for *S. stercoralis* also result in the true prevalence being underestimated [23].

Infection with hookworm or hyper-infection with *S. stercoralis* can result in anaemia, and hookworm can also present with cutaneous rash from larval migration. *Ascaris, Strongyloides* and hookworm larvae migrate to the lungs to be coughed up and swallowed, thus entering the gut where they mature. Lung-stage infection by *Ascaris* can cause pneumonia, called Loeffler’s pneumonia, while disseminated *Strongyloides* can also cause pneumonia and pulmonary haemorrhage; hookworm-associated pneumonia, ‘eosinophilic pneumonia’, is a rare manifestation [24,25,26]. *Toxocara* infections can result in a range of symptoms depending on where the larvae migrate. The migrating larvae themselves, much like in *Strongyloides* and hookworm infections, can result in a rash, or larval tracks, due to inflammation. In the eye, *Toxocara* can cause partial or total retinal detachment leading to blindness and may result in neurological symptoms if the larvae are present in the brain [18,27].

**Figure 1:**
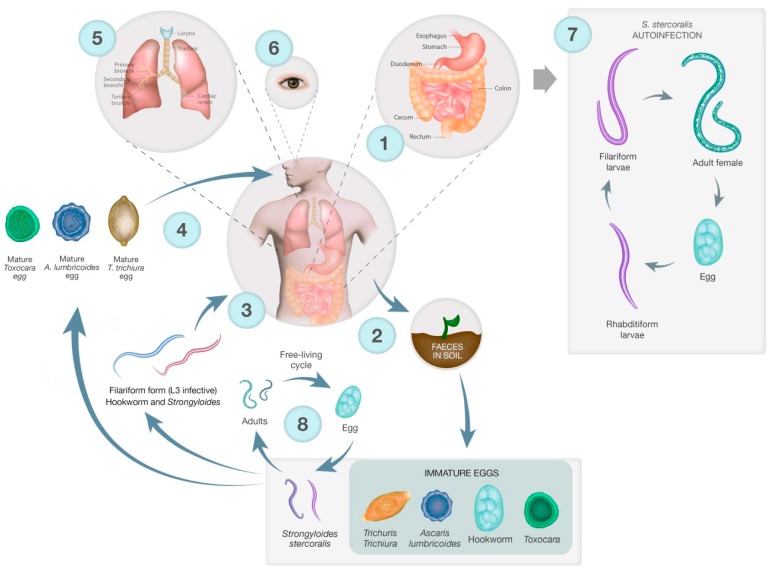
Lifecycles of soil-transmitted helminths (STH), *S. sterocoralis,* and *Toxocara.* **1.** Adult worms reside in the gastrointestinal tract (GIT). Hookworm, *A. lumbricoides,* and *S. stercoralis* adults reside in the small intestine while *T. trichiura* adults reside in the cecum and ascending colon. Female worms produce eggs which are passed in the stool of an infected person. **2.** *T. trichiura, Toxocara*, and *A. lumbricoides* eggs mature in soil but do not hatch. Hookworm eggs hatch in soil and mature into L3 hookworm larvae. *S. stercoralis* eggs hatch into rhabditiform larvae in the gut, which are then excreted via the faeces. Rhabditiform larvae then mature into infective filariform larvae or free-living adults. **3.** Infectious L3 filariform larvae of hookworm and *S. stercoralis* penetrate the skin directly, enter the circulation and migrate to the GIT after passing into the lumen of the lungs. **4.** Mature eggs of *Toxocara, A. lumbricoides,* and *T. trichiura* are swallowed by the host. The eggs hatch, releasing larvae in the GIT. *T. trichiura* larvae hatch in the small intestine and mature into adults in the colon while *Toxocara* and *A. lumbricoides* larvae penetrate the gut. *Toxocara* larvae are carried by the circulation to a variety of tissue types while *A. lumbricoides* larvae are carried to the lungs. **5.** Hookworm and *A. lumbricoides* larvae penetrate the alveolar walls and ascend the bronchial tree to the throat and are swallowed. Once they reach the small intestine the larvae mature into adults. *S. stercoralis* can also follow bronchial migration, or they can penetrate straight to the GIT. **6.** *Toxocara* larvae can be carried to any tissue type. As humans are dead-end hosts the larvae do not undergo further development once they reach these sites, they can cause local reactions, known as the disease toxocariasis. Ocular toxocariasis, where the larvae penetrate the eye, can result in blindness. **7.** *S. stercoralis* can also undergo autoinfection, where the rhabditiform larvae become infective filarial form larvae in the small intestine and penetrate the gut or perianal region. The filariform larvae can then disseminate to throughout the body. **8.** *Strongyloides* rhabditiform larvae develop into free-living adults that produce eggs from which rhabditidorm larvae hatch. Rhabditiform larvae then develop into infectious filariform larvae and penetrate a human host. The free-living cycle exists for one generation cycle only.

*Strongyloides stercoralis,* which is endemic in aboriginal communities of Australia, can be a serious roundworm infection with severe health implications due to autoinfection and dissemination. Infection in immunocompromised individuals is particularly serious, and can be fatal. Autoinfection with *S. stercoralis* occurs when the larvae produced by the adult worms cause reinfection without ever having to leave the body. In such instances there is no immune response against the migrating larvae and this can lead to hyperinfection and dissemination. Continuous reinfection with *S. stercoralis* through autoinfection can also lead to persistent infections lasting many years (Figure 1). Infections in immigrants in Australia have been found more than 20 years after moving away from an endemic area (Table 1). Disseminated strongyloidiasis occurs when the parasite is distributed throughout the body and is more commonly seen in people with impaired immune systems [28]. It can lead to abdominal pain and swelling, pulmonary and neurological complications and meningitis, depending on where the parasite is located, as well as septicaemia, a leading cause of death in *S. stercoralis* infection [28,29]. Septicaemia occurs due to migration of larvae through the gastrointestinal wall. While generally considered as a human parasite, *S. stercoralis* has also been found in non-human primates and dogs [30,31,32]. There are also haplotypes identified for this species, and grouping of haplotypes from humans with haplotypes from canids and indicates potential zoonotic transmission [33,34]. Sequencing of isolates found in dogs and humans in Cambodia found two haplotypes of *S. stercoralis* in dogs, one of which was indistinguishable from that found in humans, again indicating the potential for zoonotic transmission [30]. Dogs are an important reservoir for zoonotic infections in terms of transmission as they live in close contact with humans, increasing the likelihood of transmission when compared with other potential zoonotic hosts such as non-human primates, which have a far more limited association with humans. Australia has a high pet ownership, particularly dogs and cats, with many also living inside homes, thus providing clear potential for transmission [35].

Recent molecular analysis of hookworm species has been important for speciation and shows a different epidemiological pattern than previously thought, including a much higher prevalence of *A. ceylanicum,* which had been considered to be only a rare infection in humans [4,11,50,51,52,53]. This is due in part to the morphological similarity of eggs and larval stages between hookworm species leading to misdiagnosis, and partly due to initial erroneous assumptions of their epidemiology [53]. While dogs are thought to be the primary source of zoonotic *A. ceylanicum,* there is evidence that human–human transmission can occur [11]. Two haplotypes have been identified, with the zoonotic haplotype also identified in cats [11,54]. This has public health implications, since dogs and cats will act as reservoir hosts and will thus need to be taken into consideration for control. Dogs are also the main host for *A. caninum,* which can cause gastric enteritis in humans, and *Toxocara canis* which can cause serious eye disease often resulting in blindness, as well as neurological symptoms depending on where the parasite migrates to in the body. *T. cati,* found in cats, can also cause similar pathology. 

We review the STH, *Strongyloides* and *Toxocara* in SEA and Australia, considering their lifecycles; prevalence in SE Asia and Australia; diagnosis; and treatment and control. In addition, their zoonotic potential will be further explored.

## 2. Lifecycles

The lifecycles of the STH are shown in Figure 1, illustrating key differences in infection strategy and migration pathways. Adults of all STH species and *S. stercoralis* live in the gastrointestinal tract (GIT) and produce eggs that are excreted into the environment via the stool. STH and *S. stercoralis* require moist, warm soil to develop, largely restricting these parasites to tropical areas. Hookworm eggs hatch in the faecal mass and moult from L1 to infective L3 larvae. The L3 larvae then migrate onto vegetation, penetrate the skin, are carried via the blood to the lungs where they undergo tracheal migration, pass through to the small intestine, mature into adults and attach to the gut wall [55]. For both *T. trichiura* and *A. lumbricoides,* eggs are passed unembryonated and mature to an infectious stage after 15 days. The now infectious eggs are ingested, often due to poor hygiene and contaminated food, and hatch in the small intestine. Larvae of *T. trichiura* then mature in the small intestine into adults [56]. *A. lumbricoides* larvae penetrate the gut and undergo tracheal migration similar to hookworm larvae, and develop into mature adults once in the small intestine (Figure 1) [57].

The lifecycle of *S. stercoralis* is more complicated than other STH since it has a free-living stage in addition to the parasitic lifecycle [58]. Adults in the gut produce eggs that hatch into first-stage larvae, which have a distinctive oesophageal appearance that gives rise to the descriptive term ‘rhabditiform larva’. This first stage larva will moult to become either free-living, a dioecious adult or an infectious larva, the filariform larva. Free-living adults produce eggs that hatch as rhabditiform larvae that moult twice to become 3rd stage filariform larvae. Filariform larvae then penetrate the skin, much like hookworm L3 larvae, and migrate to the gut – this can be via the lungs and they are coughed up as occurs in the hookworm lifecycle, or direct travel to the gut. Autoinfection occurs when eggs from adult parasites hatch into rhabditiform larvae that become filariform larvae while still in the gut. The filariform larvae can complete the lifecycle in the gut, or disseminate, migrating to other organs and tissues (Figure 1). The free-living cycle for *S. stercoralis* only persists for one generation, not indefinitely as in *Parastrongyloides sp.* [59,60].

Humans are accidental hosts of *Toxocara* spp. and become infected by ingesting eggs in contaminated soil [61]. The eggs hatch in the gut and the larvae penetrate the gut and are carried by the blood to different organs where they can promote a local reaction, which is the cause of toxocariasis. Visceral and ocular migrans are the most common presentations. There is also a suggested link between seropositivity for toxocariasis and epilepsy [62].

## 3. STH and *Strongyloides* in SEA

**Figure 2:**
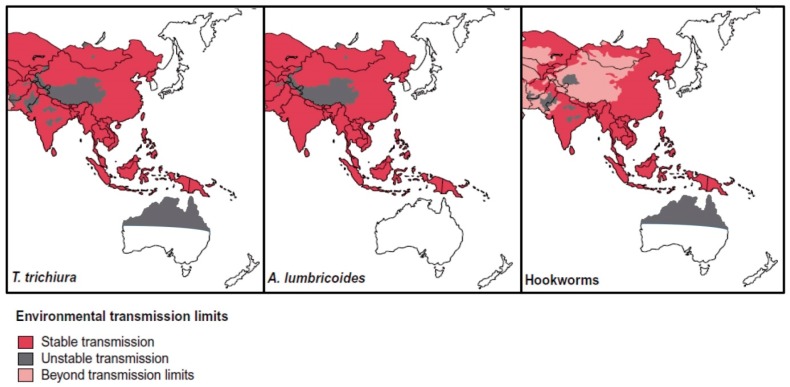
Distribution of STH in South-East Asia and Australia, modified from Brooker et al., [64].

STH infections are common in SEA, where approximately one-third of global STH cases occur, with active, stable transmission occurring in all countries of the region [63,64,65] (Figure 2). Risk factors for infection include poverty, lack of access to clean water and toilets, as well as unhygienic practices such as not washing hands [64]. 

*T. trichiura, A. lumbricoides* and hookworm are the most prevalent STH and infected individuals are often found with co-infections. Polyparasitism with STH is very common in SEA, mainly due to the shared geographical locations in tropical areas, where these worms are endemic. Additionally, the infection pathways of the STH are similar (Figure 1). Polyparasitism with STH is more likely than mono-infection in endemic areas, where co-infection with other helminths and protozoan parasites is also very high [66,67,68,69,70,71]. Because STH endemicity is quite low in Australia, data for polyparasitism is limited, although it may occur in remote Aboriginal communities [42]. 

Co-infections with parasites have been identified with increased disease status, and synergism between parasite infections. Infection intensity of helminths in co-infections also seems to differ, with hookworm infection intensity significantly increased with multiple infections [72]. Maternal infection with STH may also increase susceptibility of the unborn child to infection with STH, but it is unclear if this is due to shared environmental factors [73,74]. Co-infections may necessitate combination chemotherapy depending on drug efficacy for the infecting species.

**Figure 3:**
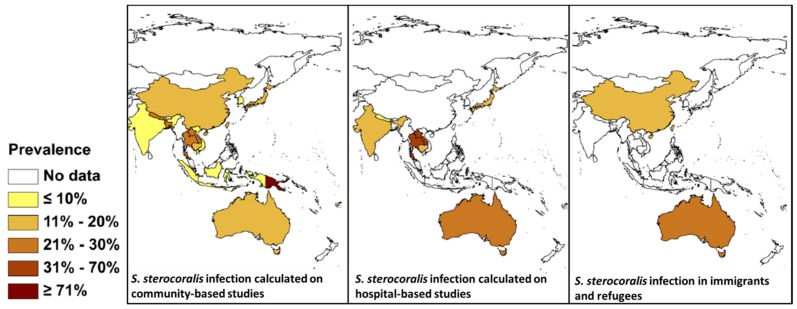
Prevalence of *S. stercoralis* in Asia and Australia based on community-based studies, hospital-based studies, and prevalence in immigrants and refugees. Modified from Schäret al., [84].

The precise global prevalence of *Strongyloides* is unknown although it is estimated to infect 30–100 million people worldwide [75]. There are also issues with diagnosis because serology can return negative results, particularly in early infections, and may not pick up disseminated cases [16]. Eggs are rarely seen in the stool; rather the rhabditiform larvae are present instead, although often in low numbers. Both copro-culture, which can be laborious, and direct smears, can be used to identify larvae [76]. Other methods include the formalin ethyl acetate sedimentation method, which has low sensitivity, immunodiagnostics, and molecular methods [77]. Molecular methods, specifically PCR, have been used to sensitively diagnose *Strongyloides* [78,79]. *Strongyloides* is endemic in SEA and there are a number of reports from countries in the region using an array of different diagnostic techniques [80,81,82,83]. A comprehensive review is available of its global distribution based on community, hospital, and refugee and immigrant surveys [84] (Figure 3). Depending on the diagnostic method used, the prevalence of *S. stercoralis* given in Figure 3 may be an underestimate due to the low sensitivity of many of the procedures used, and the low number of larvae that are excreted, even in heavy infections [77,79,84].

## 4. STH and *Strongyloides* in Australia

Australia has a low number of STH cases, largely because these worm infections can be readily controlled by good hygiene, access to safe, clean water, and the use of toilets. The overall prevalence of hookworm and *T. trichiura* in the Australian Northern Territory is quite low at 0.17% and 0.65%, respectively (Table 1), based on hospital data collected between 2002 and 2012 [42,43]. These cases were primarily detected in people admitted to hospital for reasons other than hookworm or *Trichuris* infection; thus the true prevalence may be much higher, and may follow a more focal intensity that would not be accounted for by recording state-wide or country-wide prevalence. For both species the prevalence has been reduced; with *T. trichiura*, there was a drop from 123.1 cases per 100,000 in 2002 to 35.8 cases per 100,000 in 2012 [42]. The zoonotic STHs, particularly *A. caninum* and *A. ceylanicum,* are of importance in Australia since they are found in domestic and wild canids (Table 2) [85,86]. Intestinal parasites of pigs in Australia also include *T. suis* and *A. suum,* which can infect humans, although the extent of human disease is unclear [4,87]. 

In 1918, hookworm was considered such a serious problem in North Queensland that a five-year campaign to eradicate the disease was instigated and although considered successful, hookworm infection continued to be a problem in Aboriginal communities [53,88]. The majority of published papers on STH in Australia are relatively old, with very few published in the last 10 years. Prociv and Luke [88] provide a solid review of the early history of hookworm infections in Australia. *Ascaris* spp. infection has never been very prevalent despite the requisite tropical climate and moist soil existing in Australia [89]. 

*S. stercoralis* appears to be more prevalent in Australia than the other STH, or at least, there are more published data available (Table 1) (Figure 2). The parasite is endemic in tropical regions of Australia including Queensland, the Northern Territory, Western Australia, as well as Northern NSW. It is primarily found among Aboriginal people living in remote communities, with a prevalence of >60% recorded (Table 1) [90,91,92,93,94]. This worm has persisted due to lack of attention to the disease it causes, despite the potential for high morbidity and mortality in immunosuppressed individuals [94]. Its true prevalence is unknown in Australia, and probably globally. As reported by Speare *et al* – “if you don’t look, you won’t find” [94]. As a human-only parasite, it can be readily treated with ivermectin and eliminated from the community. A retrospective study examined indigenous Australians in Central Australia who were positive for *S. stercoralis* infection and who may also have been positive for human T cell lymphotropic virus type I (HTLV-I). This virus invades adult T cells, thereby reducing the effectiveness of the immune system [29]. Of these subjects, eleven (n = 18) were tested for HTLV-I, of which seven were positive. Of those who tested positive for HTLV-I, four were never treated for *Strongyloides*, and of those who were treated, many were not treated at initial diagnosis and infection status was not checked on subsequent visits [29]. Of these eighteen patients, fifteen died from sepsis.

A complication of disseminated strongyloidiasis is secondary bacterial infection that can become systemic, and has likely contributed to mortality in at least four cases. Further studies have found high prevalence of HTLV-I in Aboriginal communities (33.6% n = 889), which may lead to more cases of hyperinfection with *S. stercoralis* in the future [118,119]. Along with immune suppression due to infection with HIV or HTLV-I, and immunosuppression due to organ transplantation, treatment with steroid drugs suppresses the inflammatory response and can also result in hyperinfection [29,119,120,121,122,123,124]. There is a clear need to increase knowledge of physicians in endemic areas, perhaps as part of a database for NTDs. Speare et al [93] advocated that *S. stercoralis* be added to the national notifiable diseases surveillance system in Australia to help combat this sadly neglected disease. Notification would bring with it greater oversight and available information to physicians, which would help lead to better management of cases, including the provision of effective treatment. To date this has not occurred and, indeed, there are no helminth diseases on the list; malaria is the sole parasite infection listed. 

## 5. Immigration Screening in Australia

Outside remote communities, it is primarily in immigrants from developing nations and returning travellers that STH cases occur in Australia (Table 1) (Figure 2). Current health screening for immigrants does not include testing for parasites and focuses on notifiable diseases such as tuberculosis and HIV/AIDS. Since STH infections are not notifiable, it is possible that there are autochthonous and returned traveller cases occurring in Australia that are not identified or reported.

Cross-sectional surveys have been performed on recent (1997–2000) and long-term immigrants to Australia in the East African and Cambodian communities in 2000 and 2002, and long-term immigrants from Cambodia [36] (Table 1). *S. stercoralis* and *T. trichiura* were identified in the East African cohort, with only *S. stercoralis* present in the Cambodian cohort. Despite having been in Australia for some years and subject to immigration screening, the high prevalence recorded, particularly in the Cambodian cohort (42%, Table 1), indicates a need to include NTDs such as STH in pre-immigration screening. *Entamoeba histolytica, Hymenolepis nana, Schistosoma* spp. (East African cohort), and *Dientamoeba fragilis* were also identified [36]. Far from being an isolated occurrence, there is a history of STH found in resettled immigrants (Table 1). An earlier study on immigrants from Laos, who had been resettled in Australia for at least 12 years prior to the survey, found *S. stercoralis* in 24% of participants (23/95) [37]. *Strongyloides* is the most commonly reported helminth infection in immigrants (Table 1). Generally *S. stercoralis* infections are asymptomatic. More severe complications from infection include eosinophilic pneumonia, malnutrition, and disseminated strongyloidiasis. 

While screening for helminth and protozoan parasites does not occur upon entry to Australia, the government does provide reimbursement for GPs who perform heath assessments within 12 months of arrival [125]. There are also state-funded refugee services in most states and territories [126,127,128,129,130]. In theory, these services could include parasitological identification. As of June 2016, 28.5% of Australians were born overseas, with five of the top 10 countries of birth in SEA (China, India, the Philippines, Vietnam, Malaysia) [131], countries with high STH endemicity. A global distribution map for STH [64] shows stable transmission occurring in SEA and Africa, origins of many immigrants coming to Australia (Figure 2 and Figure 3).

## 6. Returned Service Personnel

Another cohort for Australian STH infections are Australian army veterans, including older veterans who served in STH-endemic areas from World War II onwards (Table 2) [39,40,41,132]. *Strongyloides* is the most commonly identified helminth infection in this cohort, possibly the only STH actually considered. In Vietnam veterans, 11.6% had positive serology in 2013 for *Strongyloides*, despite serving between 1962 and 1975 [40]. This shows that the adult worms can persist for many years, as highlighted earlier for immigrants who had been long-term residents in Australia still testing positive for this disease. 

## 7. Diagnostics

### Microscopy

Stool-based microscopy remains the most common diagnostic method for STH, including in Australia, with the formol ethyl acetate sedimentation technique most commonly employed [133,134]. For *S. stercoralis,* serology is recommended, due to low and irregular numbers of larvae excreted in the stool even in heavy infections, with stool microscopy used to rule out other infections [133,135]. Diagnosis of STH in Australia is largely done on a case-by-case basis rather than by case detection. Case detection, involving diagnostic sweeps of a community, is more likely to occur as part of a research program in endemic countries. The diagnostic method used will vary. Case-by-case studies are likely to use more sensitive, albeit more laborious, diagnostics than prevalence surveys, which examine a large number or individuals and usually necessitating faster, cheaper diagnostics such as the Kato-Katz (KK) method. 

In Asia, a number of different diagnostics have been employed, often as part of specific research projects or for assessing government control programs. The main diagnostic employed is the KK method, a tool also used for diagnosis of schistosomiasis and STH in the Philippines and China. The KK procedure is cheap and easy to perform, particularly under field conditions, which are the reasons it is generally used in large-scale studies. However, the KK is known to lack sensitivity, particularly in low prevalence/intensity infections, and particularly for *Strongyloides* [136,137,138]. Additionally, hookworm eggs hatch rapidly after stool deposition, with the result that KK slides need to be prepared and examined quickly before eggs lyse [139,140]. FLOTAC is another microscopic, albeit more recent, technique that has been used in STH diagnostics, and has a higher sensitivity than the KK procedure [136,137,141,142,143,144]. The main disadvantage of FLOTAC is the length of time it takes to implement, with a single FLOTAC taking around 30 minutes to produce a result [145]. Other methods include the Baermann technique, which is based on the movement of larvae out of stool, the formalin-ether concentration technique (FECT), and coproculture [136]. *Strongyloides* has low sensitivity on stool examination so either multiple stool samples need to be examined or serology undertaken, which is the recommended diagnostic approach [77]. Other methods such as PCR, agar plate culture, and Baermann sedimentation can also be performed on stool samples, and have a higher sensitivity than microscopy [77]. Both agar plate and the Baermann technique can be time consuming. While PCR is more expensive than microscopy, agar plate, or Baermann, it achieves higher sensitivity than all of these techniques [78]. 

## 8. Immunodiagnostics

As indicated, serology is often used in Australia for diagnosis of *S. stercoralis* [133], with microscopy employed for the other STH. In general, this holds true for Asia as well. For hookworms, which as discussed earlier, have fragile eggs, immunodiagnostics can be a more sensitive detection method than faecal microscopy. However, lack of specificity, cross-reactivity, and the inability to distinguish between past and current infections are limitations of many immunodiagnostic tests. The most common immunodiagnostic methods used for STH detection are enzyme-linked immunosorbent assays (ELISAs), western blots, and ELISPOT [14,146,147]. Dipstick assays are rapid diagnostics that usually detect antibodies in blood to a target parasite. A dipstick developed for *S. stercoralis*, which is no longer available, had a similar sensitivity (91%) to the ELISA assays it was compared to and a specificity of 97.7% [148]. While serology is recommended for strongyloidiasis due to the poor sensitivity of stool examination, serology can also miss heavy infections as demonstrated by a recent fatal case from Israel of hyperinfection with *S. stercoralis* that was ELISA-negative [23]. An assortment of immunodiagnostics are available for *Strongyloides*, which have a range of sensitivities and specificities [149]. 

Dried blood spot (DBS) testing occurs by blotting blood samples onto filter paper. Samples can be collected and stored for later analysis, allowing for large numbers of samples to be collected but without requiring large amounts of storage space, and allows relative ease of collection because only a drop from a fingerprick is required. Dried blood spots have been used for molecular diagnostic tests, but can also be used for immunodiagnostics [46]. Most recently, the application of DBS was utilised in serology to diagnose *S. stercoralis* in a remote community in Northern Australia [46]. 

## 9. Molecular Diagnostics

There is a range of molecular diagnostic tests available that offer higher sensitivity than microscopy-based diagnostics, albeit with a higher price tag, and these have been reviewed elsewhere [150]. The main benefit of molecular methods is the ability to multiplex assays, that is, identify multiple species using a single assay. However, as well as being more costly than microscopy techniques, they also require specialised equipment. An exception is loop-mediated isothermal amplification (LAMP), which can be performed in the field due to much reduced equipment requirements [151,152]. However LAMP does not allow for multiplexing and to date, of the STH, has only been used to detect hookworm infections [152]. 

Molecular methods with multiplexing capabilities include conventional polymerase chain reaction (cPCR), real-time PCR (qPCR), multiparallel and tandem qPCR, and digital droplet PCR (ddPCR). Both cPCR and ddPCR are endpoint PCRs, which rely on designing primers that produce amplicons of different lengths to distinguish individual parasite species. ddPCR also uses fluorescent dyes, while real-time PCR reactions utilise fluorescent probes to distinguish between amplicons; the use of taqman probes can increase the sensitivity and specificity of an assay. Of these methods only ddPCR has yet to be used to diagnose STH infections, although it has been utilised for other parasites such as *Schistosoma* spp., detecting cell-free DNA (cfDNA) in a range of body fluids (stool, serum, urine, saliva) [153,154]. The assay provides absolute quantification and can detect very low levels of target DNA; it is also more sensitive than qPCR. While currently only providing for two channels, it is possible to multiplex the reactions for four targets by utilising different size amplicons, much as for a conventional PCR multiplex, because targets can be separated based on size. While schistosomes are blood parasites, and are thus in contact with host blood and tissues, the STH live in the gut and the potential for detection of parasite cfDNA in body fluids such as blood, urine, and saliva would likely be reduced. However, since hookworm is a blood feeder it does gain access to the host blood stream and it is possible that hookworm cfDNA would be detectable in sera. Likewise, the larvae of hookworm and *Ascaris* penetrate the alveolae of the lungs to be coughed up and swallowed, thereby reaching the gut. It is therefore possible that a saliva or sputum sample would yield cfDNA or the larvae themselves. Regardless, the use of ddPCR on stool samples will readily amplify target parasite genes with very high sensitivity. Because it confers absolute quantification and by partitioning the PCR mix (containing master mix, primers, and DNA) into ~20,000 droplets pre-amplification, it effectively means that each sample has ~20,000 technical replicates. Therefore there is no need to run samples in duplicate or triplicate for ddPCR as there is for qPCR. This can save on costs, although in practice the cost of ddPCR and qPCR is similar.

## 10. Costs of Diagnostics

The cost of a single KK slide, excluding personnel costs and stool collection costs, is US$0.30 [155]. The total cost for single and duplicate KK slides have been estimated to be $US1.73 and US$2.06, respectively, while the FLOTAC costs $2.35 [145]. In comparison, a multiplex qPCR costs $7.68. For qPCR the major costs result from DNA extraction as the qPCR assay itself costs $1.68 per sample in triplicate. A multiparallel qPCR has been costed at $1 per sample, excluding DNA extraction costs [156].

## 11. Treatment and Mass Drug Administration (MDA) of STH Infections

Mass drug administration (MDA) is a hallmark of many control programs aimed at controlling STH infections. The benzimidazoles (albendazole and mebendazole) are the most commonly used drugs for STH infection in humans, and are recommended for MDA. In the Philippines there is an annual deworming program among school-aged children. In 2003 the prevalence of STH in pre-school children was 66%, after which the Philippines Department of Health introduced the national school deworming program, The Integrated Helminth Control Program (IHCP) [157], with albendazole or mebendazole being recommended for use. The program has been successful in reducing overall prevalence at least in some areas, although MDA coverage can vary. Chemotherapy does not prevent re-infection, and once out of the school program there is no mandated MDA for STH treatment in adults. Fear of birth defects has also been recorded as a reason for refusing STH treatment by pregnant women in the Philippines [158]. School-based MDA has a compliance of >75% while community wide MDAs tend toward low compliance (25–65%) [158,159]. In some cases this is due to poor community involvement, but also because of concerns with possible side effects of the drugs. In the IHCP school deworming program, there was an increase in STH in at least one city (46.05% in 2007 to 56.60% in 2011), and the overall prevalence remained high at 45% as of 2011 [160]. Assuming 100% coverage, the problem of STH will still exist, since re-infection can occur very quickly after treatment, and STH eggs/larvae can live in the environment for several weeks to months, remaining viable for infection. Another issue is drug efficacy. While most available drugs for STH are highly efficacious for *A. lumbricoides*, there are varying efficacies for hookworm, *Strongyloides* spp. and *Trichuris* spp. Efficacy varies depending on the drug given, whether the drug is given as a single or multiple dose, and the amount given. Most programs of MDA rely on a single dose, being easier and not relying on individuals returning for treatment on multiple days. Drug efficacy for hookworm species can be difficult to untangle, as many studies do not speciate the infecting worms. There are documented differences in drug efficacy between *A. duodenale* and *N. americanus,* with mebendazole less effective against *N. americanus*, and pyrantel pamoate less effective against *A. duodenale* [161]. For all STH, drug efficacy likely varies on a regional level depending on parasite populations and treatment programs, particularly those involving MDA. 

A World Health Organization (WHO) report [162] showed a range of efficacies against STH for mebendazole, albendazole, pyrantel, levamisole, and ivermectin. For *A. lumbricoides,* efficacy was up to 100% for all drugs except ivermectin. For hookworm the highest efficacy, in terms of cure rate (CR), was achieved with levamisole (66%–100%), and for *T. trichiura* with mebendazole (45%–100%). In the same report comparing differing single doses of albendazole and mebendazole (recommended by the WHO for STH control and treatment) with multiple doses of mebendazole, multiple doses resulted in a higher CR for both hookworm and *Trichuris* spp., albeit the median CR was still around 80% [162]. However, the WHO also recommends periodic worming with albendazole or mebendazole where prevalence is >20% [163]. The aim of the WHO with regards to STH control is to carry out preventative worming in endemic countries with a prevalence of >20%. However if drug efficacy is low, particularly for hookworm and *Trichuris* spp., this approach is unlikely to decrease prevalence in the long term. 

Of relevance to treatment of STH cases in Australia and SEA, a study on immigrant populations (in Canada) found an overall reduction in intestinal parasites (STH and *Giardia*) 6 years after resettlement and treatment with thiabendazole, from 63.7% down to 21.9%; however the prevalence of *S. stercoralis* remained relatively high with a reduction from 15% to 11% [37,164]. However, since that report, ivermectin has been designated the drug of choice for *Strongyloides* spp. infection because this has a high efficacy given singly as a 200 mcg/kg dose (96% CR) or as a split 400 mcg/kg dose (98% CR) [165]. This highlights the need for combined chemotherapy when treating individuals infected with more than one species of STH.

Cure rates may be lower than reported due to insensitive diagnostics used in many drug efficacy trials, primarily stool microscopy, and variation in CR given by the same drug regimens in different trials may be due to the methods used to assess treatment success or failure. The sensitivity of diagnosis varies considerably with the more traditional microscopic diagnostics, such as the Kato-Katz procedure, lacking the sensitivity of more recently developed techniques such as real-time PCR-based diagnostics. Serological diagnosis measuring antibodies should not be used to assess CR since they will detect antibodies from previous infections for several months post-treatment, assuming 100% efficacy. Antigen-based tests are more specific but many detected antigens tend to break down quickly in the body.

In an interesting development, the bacterium *Bacillus subtilis* has been engineered to express the anthelmintic protein Cry5B, which proved lethal to *Caenorhabditis elegans* and experimental *A. ceylanicum* hookworm infections in hamsters [166]. This approach could be used by modifying ‘good’ bacteria that are safe for human ingestion. There are numerous probiotics on the market, of which the most common bacterial species are *Lactobacillus acidophilus*, *L. casei*, *Bifidobacterium lactis*, *B. bifidum,* and *Bacillus subtilis*.

Resistance to anthelmintics continues to be a concern. Currently, resistance has been reported for the human helminth, *Onchocerca volvulus* against ivermectin [167]. *N. americanus* eggs heterozygous for a β-tubulin mutation associated with resistance to benzimidazoles (albendazole, mebendazole) in *A. caninum* were recovered from a small number of individuals (n = 28) in Haiti [168]; homozygous eggs were not identified, but may be present. This raises the possibility of emerging benzimidazole resistance in human hookworm infections, as it has in veterinary hookworm. Drug resistance is common in helminths of veterinary importance where resistance has been reported against not only benzimidazoles but also levamisole, avermectins, and milbemycins (which include ivermectin) [169,170]. Resistance has not been reported for any of the zoonotic helminths. With the increase of MDA programs in STH-endemic areas, there is increasing evolutionary pressure on parasite populations, which may lead to resistance. To rigorously assess resistance, however, sensitive diagnostics will be required, and research needs to be undertaken in the search for alleles conferring resistance. Wolstenholme *et al* [170] have provided an excellent overview of drug resistance in veterinary helminths, while Vercruysse *et al* [171] consider the potential of resistance to currently-available drugs developing in human helminths.

## 12. Control Programs

STH reinfections can largely be controlled with appropriate hygiene, including washing hands after defecation, and using a toilet, as augmentation to treatment. There are several programs operating in Asia that seek to combat STH infection by using educational interventions. These include the ‘Magic Glasses’ program in China [172,173], which has now been extended to the Philippines, and the WASH (water, sanitation, and hygiene) program, which aims to provide clean water, toilets, and promoting good hygiene practices, implemented, for example, in Timor-Leste [174,175]. Helminth infection is of lesser concern in developed countries, where toilets and clean running water are available. While STH chemotherapy is effective, it does not prevent re-infections that can happen very quickly after treatment [176]. The aim of currently applied interventions is to prevent re-infection, and thus reduce the overall prevalence and eventually eliminate STH from a community. 

The Magic Glasses program targets school aged children in China to reduce prevalence of STH and to increase knowledge of STH parasites to reduce re-infections occurring [172]. The focal point of the intervention is a cartoon, produced along with other teaching aids, to teach children about STH and what they can do personally and in their homes to prevent infection, including washing hands after defecation, only using a toilet, covering food, and wearing shoes. In some schools, water tanks were provided outside toilets to facilitate handwashing. Parasite prevalence and intensity levels were assessed pre-intervention and a year later post-intervention; knowledge was also tested at these times using a questionnaire and quiz. The project was highly successful in reducing re-infection and increasing knowledge in Hunan province [172], and has been further trialled in Yunnan province. It is ongoing in the Philippines, where a new video was created to match local popular cartoons, culture and language. The program has the potential to be modified for many different cultures and areas, including Australia.

WASH for WORMS has been implemented in Timor-Leste as part of a trial where intervention villages had WASH implemented alongside mass drug administration (MDA) using albendazole, while control villages only received MDA [174,175]. Intervention villages were provided with access to clean water, the building of latrines and improving hygiene, particularly handwashing. Results of this intervention and the Magic Glasses trial in Yunnan and the Philippines are currently being formulated.

In both these types of interventions community involvement was crucial. With the Magic Glasses, one of the key components was to increase STH knowledge, focusing on school children whereas the WASH program was community-wide. Targeting only children, or any one group, may not significantly impact transmission in a community, but educational interventions in schools can be delivered by teachers, with the children and teachers taking the lessons learnt back to their families, thereby increasing community knowledge and practice [177]. Paradoxically, children often have the highest helminth infection prevalence in a community [178].

## 13. Zoonotic Roundworms

### Zoonotic Hookworms 

*Ancylostoma duodenale* and *N. americanus* are responsible for the majority of human hookworm infections. However, there are two prominent zoonotic hookworm species, *A. ceylanicum* and *A. caninum,* which can also infect humans. *A. ceylanicum,* particularly, is gaining prominence since many cases originally identified as *A. duodenale* may actually be due to *A. ceylanicum* [51]. Both *A. caninum* and *A. ceylanicum* are found in dogs, and *A. ceylanicum* infects cats (Table 2). *A. caninum* has been identified in cats, although this is uncommon (Table 2). While human infections with *A. ceylanicum* do occur, there is some discussion around whether infection with this species produces hookworm disease and morbidity (Table 1) [50,179]. Table 1 shows recent cases of *A. ceylanicum* infection in humans and animals since 2000. Conlan *et al* [179] provide a review of the historical perspective of *A. ceylanicum*. Infection with *A. ceylanicum* was confirmed by molecular methods using PCR, PCR-RFLP, sequencing, or microscopy. In Australia there have been only two recent studies, including one case study of a returned peacekeeper, identifying human infection with *A. ceylanicum.* Studies in dogs have shown that this species is present in Australia, but to a much lesser degree than *A. caninum*. In the Asia-Pacific, the main hookworm species identified is *N. americanus* (81.8%), while 18.18% harboured *A. ceylanicum*, including one individual who harboured both species [132]. An equal prevalence of *A. ceylanicum* and *N. americanus* was found in Cambodia (51.6%) [96,132]. Prevalence of hookworm in animals in Asia is also high, with dual infections of *A. ceylanicum* and *A. caninum* also occurring (Table 2). Certainly, therefore, the presence of *A. ceylanicum* in animals in Asia and Australia poses a risk to human health.

Pets or companion animals are increasingly popular and a potential source of zoonotic hookworms. Observing good hygiene practices, wearing footwear, and regular worming of pets will help prevent transmission of zoonotic hookworms to humans. However, studies on pets and their owners in Europe, which presents a similar socio-economic situation as Australia where handwashing practices, access to clean water and toilets, are similar, have shown that pet owners do not always wash their hands after handling their pets [180]. Only 15% of dog owners and 8% of cat owners stated that they always washed their hands. The same studies have also found *Toxocara* eggs on the fur of the study animals (both cats and dogs) [180,181,182,183,184]. Cats pose a particular risk for egg contamination because cat litter trays reside inside the house and are cleaned by the owner, while dog owners may also be required to pick up after their animals as well. 

The rate of pet ownership in Australia is very high. As of 2016, an estimated 62% of households had at least one pet, with dogs the most popular (39%) followed by cats (29%) [35]. Handwashing among Australia children was examined, with only 41% reported to always or mostly wash their hands after playing with animals, indicating that this is an issue in Australia as with comparable countries in Europe [185]. Pet ownership data are also available for China (25% dogs, 10% cats), South Korea (20% dogs, 6% cats), Japan (7% dogs, 14% cats) and Hong Kong (14% dogs, 10% cats) [35].

There have been few human infections in Australia and Asia identified as *A. caninum*, with the two most recent reports of human infection with this species both coming from Asia (Laos and India) (Table 2). In Australia, *A. caninum* has historically been associated with eosinophilic enteritis [186,187,188,189,190,191,192], although there are limited reports on this condition since the mid-1990s.

## 14. Toxocara 

Toxocariasis is caused by the migration of *Toxocara* larvae to various tissues, causing visceral larva migrans. Traditionally the species involved are *T. canis* and *T. cati* nematodes of dogs and cats respectively, with the latter most likely to be involved in human disease [193]. Other *Toxocara* species may also cause infection such as *T. malaysiensis,* which is found in Malaysia, Vietnam, and China, although its potential to infect humans at this point is unknown [193,194,195]. *Toxocara malaysiensis* also infects cats. Stray dogs and cats are a primary source of infection in developing countries, while high pet ownership in developed countries means that pets are the main source of infection there. Soil samples taken from playgrounds in Malaysia found that 95.7% of samples tested had *Toxocara* eggs, and 88.3% had hookworm, showing very high contamination of the local soil with parasite eggs infectious to humans [196]. 

The most serious result of infection with *Toxocara* is ocular toxocariasis, which can lead to blindness. Humans are dead-end hosts; the parasite larvae migrate to many tissues, and while they do not develop further they can cause granulomas and inflammation in the tissues they reside in [197]. 

In Australia *T. canis* has long been known to exist with high prevalence found in dogs and in environmental samples [198,199]. There are very few reports of recent surveys of animals, environmental samples, or humans in Australia. In the 1990s the *T. canis* prevalence in the general population of Australia was 5.7% while in Aboriginal communities it ranged from 11.1%–43% [47]. Historical infections reported included serological examination of patients with ocular symptoms in Victoria (3.86% n = 621) [200]; 7% (n = 660) seroprevalence was recorded in healthy blood donors from the Australia Capital Territory [201]. More recently 21% (n = 29) prevalence was reported in a remote Aboriginal community in the Northern Territory [47]. 

## 15. *Ascaris suum*

It was originally thought that *A. suum* infected humans only rarely, but molecular tools have shown that the two species (*A. lumbricoides* and *A. suum*), which are morphologically identical, may in fact be one species and *A. suum* represents a haplotype of *A. lumbricoides*. It has been recognised that the haplotype, *A. suum,* can cause human infections; and has also been found in non-human primates [15,202]. A study in Japan sequenced the ITS1 region of *Ascaris* derived from humans and pigs, finding that 3 of 9 isolates derived from humans were identical to those derived from pigs [6]. A phylogeny study performed in China utilising the mitochondrial genes *cox1* and *nad1,* and found a high level of gene flow between human- and pig-derived *Ascaris*, as well as indicating the presence of 20 haplotypes based on the *cox1* gene and 26 based on the *nad1* [203]. There is also evidence from China of hybrid forms of *Ascaris* [204]. In India PCR-RFLP was performed on dog stools identifying the presence of *A. lumbricoides* eggs [205].

*A. suum* has been identified in pigs in Australia; however, limited molecular work has been done to characterise the genotypes. Due to control measures in pigs it is estimated that only 3% of pigs in Australia harbour *Ascaris* [206]. There is also limited information on *A. suum* human infections in Australia. Two cases of *Ascaris* infection in Tasmania were thought to be *A. suum* based on morphology of mouthparts, and based on history of contact with pigs; no molecular tools were utilised [207].

Worldwide, 800 million people are estimated to be infected with *Ascaris*. A number of prevalence studies have been done in developing countries using both microscopy, primarily KK, and molecular techniques [66,208,209,210]. Speciation, or the presence of haplotypes, is rarely looked for in prevalence studies; it is therefore difficult to calculate the number of *Ascaris* infections due to pig-derived worms.

## 16. *Trichuris suis* and *T. vulpis*

Pigs and canines are the primary hosts of *T. suis* and *T. vulpis*, respectively. Human infections with both species have been recorded, as have hybrids of *T. suis* and the human species *T. trichiura* [5,9,10,13]. In Thailand, both dogs and humans harbour *T. vulpis* and *T. trichiura,* indicating the likelihood of zoonotic transmission occurring for both species [5]. However, dogs in India appear to only carry *T. trichiura*, while further human infections with *T. vulpis* have occurred in North America and Africa [13,205,211]. 

Genetic analysis of *Trichuris* species in humans, pigs, and non-human primates indicates that there may be several genotypes of *Trichuris* circulating in humans and animals [212,213,214]. Sequencing of pig and human derived *Trichuris* indicates that they are separate species [215], unlike *A. suum* discussed above, which is considered to be the same species as *A. lumbricoides*. Experimental infections of humans with *T. suis* documented the previously undocumented symptoms of infection which included flatulence, diarrhoea, and upper abdominal pain, although these symptoms regressed over time, after repeated exposure to eggs, to subclinical symptoms [216].

## 17. Helminth Therapy

While not all STH have as severe consequences as *Strongyloides*, it is clear that treatment of STH is needed in infected individuals. However, it has also been suggested that intestinal worms may actually be beneficial to human health, particularly to bowel health and diseases of hypersensitivity. This is usually described as part of the hygiene hypothesis, linking ‘cleaner’ living conditions with higher levels of allergic disease, and now extended to inflammatory disorders such as inflammatory bowel disease (IBD) [217]. Helminth parasites are known to modulate the host immune system and it is thought that by doing so for self-benefit, this may cause a bystander effect on other immune-related diseases [218,219]. 

There have been clinical trials with hookworm and *Trichuris* for treatment of a range of syndromes, including coeliac disease, asthma, allergies, and psoriasis [220]. Hookworm and *Trichuris* eggs are available online through companies such as Wormswell [221], which provides *N. americanus* eggs, and Tanawisa, which provides *T. suis* (porcine *Trichuris* species) (TSO) [222] eggs. The link between asthma regulation and helminths has been studied for some time, although concrete significance in trials has yet to eventuate, and exact mechanisms of action are unclear [223]. Recent trials for asthma, where individuals were experimentally infected with hookworm, indicated some improvement between groups with hookworm and those without, but the results were not significant [220]. A study in Uganda showed maternal hookworm infections decreased the risk of childhood eczema [224]. Australian studies of hookworm therapy largely focus on coeliac disease, which has had mixed results with null or positive associations; any causal link between allergies and parasite infection has not been shown [225,226]. Gut microbiota may also play a role, although it is still unclear how parasites and the microbiota interact with each other [227]. While *T. suis* is primarily a parasite of pigs, it can establish patent infections in humans [9,10,228] and treatment with TSO has been linked to improvement of psoriasis [229]. 

So, whereas STH and *Strongyloides* are generally thought to be detrimental to health, the presence of a few worms might be beneficial, although more research needs to be performed to confirm this. Because of the risk of self-infection with helminth eggs, most current work aimed at clinical trials is focused on the isolation of particular components in worm secretions that provide beneficial protection or immune modulation of IBD diseases [230,231]. 

## 18. Discussion and Conclusion

While STH infections are generally low in Australia, they are still endemic and of significant health importance in remote Aboriginal communities, particularly *S. stercoralis,* which can be fatal in immunocompromised people. Limited data are available for hyperinfection and mortality due to strongyloidiasis in the Asia-Pacific area, but in Australia, infection has led to fatalities, which, in such a resource-rich country, is alarming and unacceptable. Part of the problem may be due to a general lack of awareness of worm infections, which have a low prevalence in the country as a whole. A physician in an urban area may never see, diagnose or treat a helminthiasis case [93,94]. In Aboriginal communities, where the prevalence of strongyloidiasis can be quite high (0.25%–59.5%) [90,91,92,94,232,233], the very high cost of healthcare delivery limits available services, and physicians may only be resident for a short time so that knowledge about STH and *S. stercoralis,* in particular, may not be being passed on or recorded in the hospital system. Adding STH, particularly especially *S. stercoralis,* to the national notifiable disease system would help increase knowledge and provide access to more information about the appropriate handling of these parasitic worm infections. 

High prevalence of STH in refugees and immigrants from endemic areas, particularly the Asia-Pacific, where the majority of immigrants to Australia now originate, highlights a need for better and more comprehensive health screenings of these groups that include parasite diagnosis and treatment. This holds true for army veterans and current members who have served, or are serving, in STH-endemic areas, including long-term follow-ups in case of poor drug efficacy or ongoing autoinfection in the case of *S. stercoralis*. The Asia-Pacific area has particularly high endemicity for STH and, with so much movement between Asia-Pacific and Australia, better understanding and treatment of STH infections are key from a public health perspective. 

## Figures and Tables

**Table 1:** Prevalence of soil-transmitted helminths diagnosed in immigrants, refugees, ADF^#^ personnel, and returned travellers in Australia since 2000.

Years Sampled	Reference	Status	Country of Origin	Parasite Species	Prevalence	Diagnostics
2000, 2002	[36]	Immigrant	East Africa Cambodia	*S. stercoralis* *T. trichiura* *S. stercoralis* Hookworm spp.	11% (n = 124) 4% (n = 124) 42% (n = 230) 1.96% (n = 230)	Faecal samples (method unclear) Serology (method unclear)
7–20 years after resettlement	[37]	Immigrant	Laos	*S. stercoralis*	24.21% (n = 95)	Faecal microscopy Strongyloides serology
2–52 years after resettlement 1998–2005	[38] *	Immigrant	Fiji (1), SEA (5), China (1), Sri Lanka (1), India (2), Seychelles (2), Ethiopia (2), Russia (1), Italy (1), Greece (1)	*S. stercoralis*	100% (n = 17) *	Faecal microscopy Strongyloides serology
1998–2005	[38]*	Returned travellers	Papua New Guinea (1), Vanuatu (1), SEA (7), Africa (2)	*S. stercoralis*	100% (n = 11) *	Faecal microscopy Strongyloides serology
2004	[39]	Returned ADF ^#^ member	Solomon Islands	*A. ceylanicum*	100% (n = 1)	Harada-Mori culture, direct faecal smear
Served 1962–1975 2010	[40]	ADF veterans	Vietnam	*S. stercoralis*	11.6% (n = 249)	Faecal microscopy ELISA
2006–2007	[41]	RAMSI personnel ***	Solomon Islands	*S. stercoralis*	100% (n = 14) *	Faecal microscopy, Serology (ELISA)
2002–2012	[42]	Residents Northern Territory	Australia	*T. trichiura*	0.65% (n = 63,668) **	Wet mount microscopy, Concentration method
2002–2011	[43]	Residents Northern Territory	Australia	Hookworm	0.17% (n = 64,691) **	Wet mount microscopy, Concentration method
2004–2008	[44]	Immigrants	Burma	*S. stercoralis*	26% (n = 156)	Serology
2002	[45]	Immigrants	Cambodia	*S. stercoralis*	36% (n = 234) *	ELISA, faecal microscopy
2010–2011	[46]	Residents Northern Territory	Australia	*S. stercoralis*	16.5% (n = 124) pre-treatment 12% (n = 30) post-treatment	Serology (NIE ELISA, NIE-DBS-ELISA)
2000–2006	[29]	Residents	Australia	*S. stercoralis*	100% (n = 18) *	Faecal microscopy, serology
1994–1996	[47]	Residents	Australia	*T. canis* *S. stercoralis* *S. stercoralis*	21% (n = 29) 28% (n = 29) 19% (n = 314)	Serology Serology Formol-ether
	[48]	Immigrant	Laos	*S. stercoralis*	Single patient	Larvae in sputum
2010–2011	[49]	Immigrants Residents Residents	Australia	*N. americanus* *A. ceylanicum* *A. duodenale*	(n = 5/227) **** (n = 2/227) (n = 4/227)	PCR Sequencing

**^#^** Australian Defence Force (ADF); * retrospective review of positive cases; ** faecal samples; *** Regional Assistance Mission to Solomon Islands; **** faecal samples from individuals with a documented history of gastrointestinal disorders.

**Table 2:** Reported prevalences of *A. ceylanicum* human and animal infections in studies conducted in Australia and Asia since 2000.

Ref	Year	Country *	Human/Animal	Prevalence % (Total no.)	Species	Diagnostic
[95]	-	Taiwan	Human	Single patient	*A. ceylanicum*	Morphology
[69]	2009	Laos	Human	17.6% (n = 17) 82.4% (n = 17)	*A. ceylanicum* *N. americanus*	Nested PCR
[96]	2012	Cambodia	Human	51.6% (n = 124) 51.6% (n = 124) 3.2% (n = 124)	*A. ceylanicum* *N. americanus* *A. duodenale*	Microscopy, PCR
			Dog	94.4% (n = 90) 8.9% (n = 90) 1.1% (n = 90)	*A. ceylanicum* *A. caninum* *N. americanus*	Microscopy, PCR
[49] ^#^	2010–2011	Australia	Human	0.88% (n = 227) 1.76% (n = 227) 1.76% (n = 227)	*A. ceylanicum* *A. duodenale* *N. americanus ^a^*	PCR
[97]	-	China	Dog Cat Human	3 (n = 254) 5 (n = 102) 14 (n = 14)	*A. ceylanicum*	PCR sequencing
[98]	-	Malaysia	Dog	52% (n = 224) 48% (n = 224)	*A. ceylanicum* *A. caninum*	FECT, PCR
[99]	2007–2010	Malaysia	Cat	29.5% (n = 543)	*A. ceylanicum*	Microscopy
[100]	2009–2011	Malaysia	Human	87.2 (n = 47) 23.4 (n = 47)	*N. americanus* *A. ceylanicum*	Microscopy, PCR
[101]	2013	Malaysia (Chinese)	Human	Single patient	*A. ceylanicum*	Microscopy
[11]	2009–2011	Malaysia	Human	12.8% (n = 634) 76.6% (n = 634) 10.6% (n = 634)	*A. ceylanicum* *N. americanus* *Both species*	Microscopy, PCR
			Cats and dogs	52% (n = 105) 46% (n = 105)	*A. caninum* *A. ceylanicum*	Microscopy, PCR
[102]	-	Myanmar	Human	72.72% (n = 11) 27.27% (n = 11)	*N. americanus* *A. ceylanicum*	PCR sequencing
[103]	2004–2005	Australia	Dog	6.5% (n = 92) 70.7% (n = 92) 4.3% (n = 92) 2.2% (n = 92)	*A. ceylanicum* *A. caninum* *A. caninum + A. ceylanicum* *A. caninum + U. stenocephala*	Microscopy, PCR-RFLP
			Cat	30% (n = 10)	*A. caninum*	
[12]	2011–2013	Thailand	Human	60% (n = 10) 30% (n = 10) 10% (n = 10)	*N. americanus* *A. ceylanicum* *A. duodenale*	PCR sequencing
[86]	>2007	Australia	Wild dog	100% (n = 26) 11.5% (n = 26)	*A. caninum* *A. ceylanicum* + *A. caninum*	Microscopy, PCR
			Dog scat	65.31% (n = 89) 71.43% (n = 89) 38.78% (n = 89)	*A. ceylanicum* *A. caninum* *A. caninum + A. ceylanicum*	
[39]	2004	Australia (Solomon Islands)	Human	Single patient	*A. ceylanicum*	Microscopy
[51]	2004–2005	Thailand	Dog	77% (n = 229) 9% (n = 229) 14% (n = 229)	*A. ceylanicum* *A. caninum* Both species	PCR
			Human	71.43% (n = 204) 28.57% (n = 204)	*N. americanus* *A. ceylanicum*	
[104]	2008	India	Dog	50.46% (n = 325) 51.92% (n = 104) 33.65% (n = 104) 15.38% (n = 104)	Hookworm spp. *A. caninum* *A. ceylanicum* *A. caninum + A. ceylanicum*	Microscopy, PCR-RFLP
[105]	2000	India	Dog	36% (n = 101) 38% (n = 101)	*A. caninum* *A. caninum + A. braziliense*	Microscopy, PCR-RFLP + sequencing
[85]	2011	Australia	Dog	96.4% (n = 84) 16.67%(n = 84) 14.0% (n = 84)	*A. caninum* *A. ceylanicum* *A. caninum + A. ceylanicum*	Microscopy, PCR
[106]	2013–2014	Malaysia	Dog	29.6% (n = 227) 6.6% (n = 227)	*A. ceylanicum* *A. caninum*	FECT, PCR
			Soil samples	14.3% (n = 126) 2.4% (n = 126)	*A. ceylanicum* *A. caninum*	
			Cat	29.6% (n = 152) 6.6% (n = 152)	*A. ceylanicum* *A. caninum*	
[107]	2015	Japan (Lao)	Human	Single patient	*A. ceylanicum*	Microscopy, PCR
[108]	2013–2015	India	Human	100% (n = 143) 16.8% (n = 143) 8.4% (n = 143)	*N. americanus* *A. caninum* *A. duodenale*	
			Dog	27.9% 76.4%	*A. ceylanicum* *A. caninum*	PCR-RFLP
			Soil samples	60.2% (n = 78) 29.4% (n = 78) 16.6% (n = 78) 1.4% (n = 78)	*A. ceylanicum* *A. caninum* *A. duodenale* *N. americanus*	
[109]	2014	Thailand	Dog Cat	33.0% (n = 197) 58.46% (n = 180)	*A. ceylanicum*	Microscopy, PCR
[110]	2014	France (Myanmar)	Human	Single patient	*A. ceylanicum*	Microscopy, PCR
[111]	2014	Vietnam	Dog	54.3% (n = 94) 33% (n = 94) 12.7% (n = 94)	*A. ceylanicum* *A. caninum* *Both species*	PCR-RFLP, PCR (cox1)
[112]	2014	China	Cat	40.8% (n = 112) 59.2% (n = 112) 20.4% (n = 112)	*A. ceylanicum* *A. caninum* *Both species*	Microscopy, PCR
[113]	-	India	Human	95% 15% 5%	*N. americanus* *A. duodenale* *A. ceylanicum*	PCR-RFLP
[114]	2005	Thailand	Human	92% 4% 2% 2%	*N. americanus* *A. ceylanicum* *A. duodenale* *N. americanus* + *A. ceylanicum*	KK, PCR
[115]	2008	Lao	Human	5.91% 2.46% 1.48% 0.49%	*N. americanus* *A. duodenale* *A. caninum* *A. ceylanicum*	KK, PCR
[99]	2007–2010	Malaysia	Feral cats	29.5% (n = 251)	*A. ceylanicum*	Microscopy of adults (staining paracarmine)
[116]	-	Lao	Feral cats	69% (n = 55)	*A. ceylanicum*	Microscopy of adults (staining Mayers carmine)
[117]	-	Taiwan	Human	Single patient	*A. ceylanicum*	Method unclear. Adult identification.

* Origin of infection in brackets if not the same as the country of detection; ^#^ faecal samples from individuals with a documented history of gastrointestinal disorders; ^a^ individuals infected with *N. americanus* were refugees from Sierra Leone and Sudan, likely to be acquired in those countries.

## List of Abbreviations

STH	soil transmitted helminths
SEA	South-East Asia
DNA	deoxyribonucleic acid
qPCR	quantitative real-time polymerase chain reaction
cPCR	conventional polymerase chain reaction
ddPCR	digital droplet polymerase chain reaction
RFLP-PCR	restriction fragment length polymorphism polymerase chain reaction
Cox 1	cytochrome oxidase 1
LAMP	Loop-mediated isothermal amplification
GIT	gastrointestinal tract
WASH	water, sanitation, and hygiene
MDA	mass drug administration
NTDs	neglected tropical diseases
DALYs	disability adjusted life years
YLDs	years lived with disability
HTLV-I	human T cell lymphotropic virus type I
CR	cure rate
IHCP	the integrated helminth control program
WHO	World Health Organization
IBD	inflammatory bowel disease
KK	Kato-Katz
ELISA	enzyme linked immunosorbent assay
DBS	dried blood spot
TOS	*Trichuris suis* eggs
